# Psychometric Properties of the Korean Version of the Mental Health Professionals Stress Scale

**DOI:** 10.3389/fpsyt.2021.685423

**Published:** 2021-08-30

**Authors:** Eun Sol Lee, Vin Ryu, Ji Hyun Lee, Hyeon Hong, Hyeree Han, Subin Park

**Affiliations:** ^1^Division of Mental Health Research, Mental Health Research Institute, National Center for Mental Health, Seoul, South Korea; ^2^Mental Health Research Institute, National Center for Mental Health, Seoul, South Korea

**Keywords:** mental health professionals, stress, depression, anxiety, clinical psychologists, nurses, social workers

## Abstract

**Background:** Job stress of mental health professionals can have a negative impact on them, particularly their psychological health and mortality, and may also affect organizations' and institutions' ability to provide quality mental health services to patients.

**Aim:** This study aimed to: (1) investigate the validity and reliability of the Korean Mental Health Professionals Stress Scale (K-MHPSS), (2) develop K-MHPSS cut-off points to measure clinical depression and anxiety, and (3) examine whether specific stressors vary by area of expertise.

**Methodology:** Data were collected *via* an online survey over 3 months, from August to October 2020. An online survey using a survey website was administered to volunteers who accessed the link and consented to participate. Data from 558 participants (200 clinical psychologists, 157 nurses, and 201 social workers) were included in the final analysis. Confirmatory and exploratory factor analyses were conducted to examine the factor structure of the K-MHPSS; concurrent validity of the scale was determined by analyzing correlation; internal consistency was determined by Cronbach's alpha coefficient. In addition, ROC curve analysis and Youden's index were used to estimate optimal cut-off points for K-MHPSS; one-way ANOVA was performed to investigate the difference among the three groups.

**Results:** The seven-factor model of the original scale did not be replicated by Korean mental health professionals. The K-MHPSS had the best fit with the six-factor model, which consists of 34 items. Concurrent validity was confirmed, and overall reliability was found to be good. The K-MHPSS cut-off points for depression and anxiety appeared to slightly different by professional groups. Furthermore, nurses and social workers showed significantly higher total scores compared to clinical psychologists, and there are significant differences in subscale scores among professionals.

**Conclusion:** The Korean version of the MHPSS has appropriate psychometric properties and can be used to assess the occupational stress of mental health professionals. It can also serve as a reference point for screening clinical level of depression and anxiety in mental health professionals.

## Introduction

Mental health professionals' job stress and mental suffering are occasionally underestimated because they have more psychiatric information and knowledge than the general public. In addition, sometimes, mental health professionals themselves demonstrate a negative attitude toward mental disorders (1) or hesitate to seek help for their problems (2). However, given the nature of their jobs, mental health professionals often attend to patients and clients with psychiatric problems for prolonged periods, which consequently exposes them to emotional exhaustion and mental stress (3).

Previous studies have shown that job stress has detrimental effects on mental health professionals' mental health (4–6). For instance, job stress reported by nursing professionals working in mental health services was significantly related to depression (5). Further, association between job stress and burnout was observed among mental health professionals (7–9). Burnout is characterized as a syndrome of emotional exhaustion and cynical attitude toward clients, which is often experienced by human-service workers (10). A meta-analysis study estimated that 40% of mental health professionals experience emotional exhaustion, a key dimension of burnout (11). Moreover, a recent study showed that the effect of job stress on psychological problems was mediated by burnout (6). Specifically, nurses' job stress can cause mental health problems, such as depression and anxiety, through the mediation of emotional exhaustion. Compared to nurses in other specialties, psychiatric nurses not only experience emotional exhaustion more frequently but also show a markedly higher risk of suicide (12).

Stressors experienced by mental health professionals are also known to impact their job satisfaction, absenteeism, job performance, and turnover (13–16). A study of community mental health professionals in Korea also revealed that job stress influences turnover intention (17, 18). Further, poor work attitudes such as turnover intention, turnover, and absenteeism resulting from stress deteriorate the quality of patient and client service, while adding to organization and institution losses by increasing the cost and effort to train and foster new personnel (19). Given previous findings, job stress of mental health professionals can affect individuals, particularly their psychological health and mortality, and may also affect the organizations' and institutions' ability to provide quality mental health services to patients. Consequently, identifying mental health professionals' job stress and providing effective interventions is crucial.

Among professionals who provide mental health services, the key stressors differ across areas of expertise. Past studies have revealed the stressors specific to each mental health discipline. For clinical psychologists, the major stressors are workload, lack of resources, poor management quality, conflicting roles and relationships with other professionals, lack of work–life balance, and treating clients with chronic and complex mental health problems (20–22). One study reported that professional self-doubt is the greatest stressor for clinical psychologists (23); that is, doubts about the effectiveness of therapy they provide and perceived limitations in their competence can cause stress. Moreover, since therapists must immerse themselves in client sessions and engage in an emotional relationship with clients to provide effective therapy, it is a fundamentally stressful and emotionally burdening activity (23). Since providing therapy is the main work description of clinical psychologists, factors related to therapy are anticipated to be greater stressors for clinical psychologists than for professionals in other fields.

Stressors experienced by mental health nurses include lack of resources, workload, client-related difficulties, and organizational structures and processes (24–26). A qualitative study on mental health nurses presented detailed descriptions of these stressors (27). Nurses are put in charge of clinical work, management, and administrative work because of understaffing; are exposed to patients' violent and aggressive behaviors when organizations fail to establish appropriate measures; and lack support and recognition from management for their positive practice. They experience all these while being placed in a critical work environment with no leniency for errors, thus elevating their stress. Such stressors seem to have a larger impact on nurses than other mental health professionals because they work in an environment that places them in close contact with patients for prolonged periods (28).

Mental health social workers experience stress from lack of resources and staffing, relationships and conflicts with other professionals, workload, and organizational structures and processes (29, 30). In addition, being undervalued at work, having limited latitude in decision-making, and ambiguous roles as social workers in mental health service are also stressors for this group (31). Social workers play a wide-ranging and comprehensive role in providing mental health services, from direct client care to management; thus, role conflicts and ambiguity undermine their job satisfaction (32).

In addition to mental health professionals' stressors differing by job characteristics, their work is distinguished from other jobs, including general physical health workers' jobs; therefore, general job stress scales or job stress scales developed for healthcare providers cannot accurately detect mental health professionals' stressors (33). In Korea, few studies have measured mental health professionals' stress, and some studies did utilize the Occupational Stress Scale for Korean Employees (34), which is a valid and reliable scale developed to evaluate employees in various fields, including health and social work, but the scale lacks items specific to mental health and thus is not appropriate for mental health professionals in Korea. Another commonly used scale is the Occupational Stress Measurement for Psychiatric Nurses (35), but it contains 88 items and is thus time-consuming, which limits its utility for large-scale studies. Moreover, since it is specifically tailored to mental health nurses, its use is limited with other types of mental health professionals.

The Mental Health Professionals Stress Scale (MHPSS) was developed by Cushway et al. (33) to measure stressors experienced by mental health professionals. The items were based on stressors affecting mental health professionals identified in previous studies. The scale consists of seven subscales, each containing six items for a total of 42 items. The MHPSS was validated with 154 clinical psychologists and 111 psychiatric nurses and has been used to identify stressors in studies of clinical psychologists, psychiatric nurses, and mental health social workers (30, 36, 37). In Korea, the MHPSS was validated for use with counselors in a study by Choi and Lee (38), and a factor analysis showed that a four-factor, 36-item structure was appropriate for the purpose. This study limited the sample to counselors; therefore, whether the finalized factor structure and items apply to other mental health professionals is unknown. Thus, the present study aimed to validate the MHPSS for use with mental health professionals in Korea, including clinical psychologists, mental health nurses, and mental health social workers so that the tool can be used to identify stressors in these professions.

The study objectives were to: (1) investigate the validity and reliability of the Korean MHPSS by conducting factor and correlation analyses and examining internal consistency to determine whether the tool has adequate reliability; (2) develop MHPSS cut-off points to screen for clinical depression and anxiety that can be used by mental health professionals to determine their stress level and screen for depression and anxiety, adding to its clinical utility; and (3) examine whether specific stressors vary by area of expertise, and test the relative contributions of stressors to psychological problems. This is particularly helpful to establish a framework for prevention and intervention strategies for mental health professionals' mental health issues.

## Materials and Methods

### Study Design

To validate the MHPSS for mental health professionals in Korea, we collected data *via* an online survey over 3 months, from August to October 2020. Notifications outlining the study purpose and participant eligibility were posted to relevant mental health expert associations and academic societies. An online survey using a survey website was administered to volunteers who accessed the link and consented to participate.

### Participants

The inclusion criterion was professionals working in a mental health-related organization, namely clinical psychologists, nurses, and social workers, whose work directly involved patients and clients. A total of 571 volunteers participated in the online survey. After excluding 13 duplicate responses, non-mental health experts, or professionals working in other areas of mental health, data from 558 participants (200 clinical psychologists, 157 nurses, 201 social workers) were included in the final analysis (Table 1). This study was approved by the Institutional Review Board of the National Center for Mental Health (116271-2020-26).

**Table 1:** Participants' socio-demographic characteristics (*N* = 558).

		**Clinical psychologist *n* (%)**	**Nurse *n* (%)**	**Social worker *n* (%)**	**Total *n* (%)**	**χ^2^**
Total		200 (35.9%)	157 (28.1%)	201 (36.0%)	558 (100%)	
Gender	Male	33 (16.5%)	15 (9.6%)	54 (26.9%)	102 (18.3%)	18.35^***^
	Female	167 (83.5%)	142 (90.4%)	147 (73.1%)	456 (81.7%)	
Level of education	Bachelor	14 (7.0%)	101 (64.3%)	136 (67.6%)	251 (45.0%)	185.72^***^
	Masters	167 (83.5%)	47 (29.9%)	62 (30.9%)	276 (49.4%)	
	PhD	15 (7.5%)	8 (5.1%)	3 (1.5%)	26 (4.7%)	
	Other	4 (2.0%)	1 (0.7%)	0 (0.0%)	5 (0.90%)	
Work experience	<1 yr	26 (13.0%)	5 (3.2%)	8 (4.0%)	39 (7.0%)	105.70^***^
	1–10 yr	163 (81.5%)	79 (50.3%)	152 (75.6%)	394 (70.6%)	
	11–20 yr	10 (5.0%)	49 (31.2%)	36 (17.9%)	95 (17.0%)	
	21 yr+	1 (0.5%)	24 (15.3%)	5 (2.5%)	30 (5.4%)	
Office hours (per week)	11–20 h	17 (8.5%)	1 (0.6%)	4 (2.0%)	22 (3.9%)	42.24^***^
	21–30 h	8 (4.0%)	1 (0.6%)	0 (0.0%)	9 (1.6%)	
	31–40 h	106 (53.0%)	93 (59.2%)	108 (53.7%)	307 (55.0%)	
	41–50 h	51 (25.5%)	59 (37.6%)	77 (38.3%)	187 (33.5%)	
	50 h+	18 (9.0%)	3 (1.9%)	12 (6.0%)	33 (5.9%)	
Income level	0–1.9 million KRW	16 (8.0%)	4 (2.5%)	9 (4.5%)	29 (5.2%)	40.67^***^
	2–2.9 million KRW	105 (52.5%)	80 (51.0%)	151 (75.1%)	336 (60.2%)	
	3–3.9 million KRW	54 (27.0%)	57 (36.3%)	35 (17.4%)	146 (26.2%)	
	≥4 million KRW	25 (12.5%)	16 (10.2%)	6 (3.0%)	47 (8.4%)	
Type of work	Psychotherapy/counseling	144 (31.0%)	39 (10.7%)	100 (17.8%)	283 (20.3%)	632.90^***^
	Psychological assessment	162 (34.9%)	11 (3.0%)	26 (4.6%)	199 (14.3%)	
	Rehabilitation	14 (3.1%)	26 (7.1%)	75 (13.3%)	115 (8.3%)	
	Education (family, caregiver, etc.)	53 (11.4%)	73 (20.1%)	108 (19.2%)	234 (16.8%)	
	Case management	41 (8.8%)	70 (19.2%)	164 (29.1%)	275 (19.8%)	
	Patient care/nursing	0 (0.0%)	99 (27.2%)	1 (0.2%)	100 (7.2%)	
	Supervision	27 (5.8%)	33 (9.1%)	59 (10.5%)	119 (8.6%)	
	Other	23 (5.0%)	13 (3.6%)	30 (5.3%)	66 (4.7%)	

*** *p < 0.001*.

Table 1 shows participant characteristics for the full sample (*n* = 558) and the sub-groups (clinical psychologists, nurses, and social workers). A chi-squared test of independence was conducted, and overall sociodemographic characteristics showed a significant difference (see Table 1). Participants' mean age was 33.58 (±4.35) years for clinical psychologists, 36.98 (±8.55) years for nurses, and 32.83 (±5.99) years for social workers; the percentage of women was higher in all three groups (83.5% for clinical psychologists, 90.4% for nurses, 73.1% for social workers). While social workers and nurses predominantly had a bachelor's degree, clinical psychologists predominantly had a master's degree, which is a major qualification needed for clinical psychologist certification. Most participants in all three groups had a career length between one and 10 years (81.5% for clinical psychologists, 50.3% for nurses, 75.6% for social workers). The main work was therapy/counseling (31%) and psychological evaluation (34.9%) for clinical psychologists, patient care (27.2%) and patient/caregiver education (20.1%) for nurses, and case management (29.1%) and patient/caregiver education (19.2%) for social workers.

### Translation

The original English version of MHPSS was translated into Korean by two individuals with a master's degree in psychology and one with a master's degree in occupational therapy master with English proficiency; a bilingual psychology professor reviewed and revised the translation. One bilingual professional back-translated the original translation, and then the back translation was modified to accurately convey the source text meanings by a psychiatrist and researchers with master's degrees in psychology and occupational therapy. Conflicts were resolved through discussion. Then, three certified clinical psychologists again reviewed the translation to check for any inappropriate Korean culture context before finalizing the translated version.

### Measurement

#### Background Information

Sociodemographic characteristics included age, sex, education level, length of career, weekly work hours, income, and type of work.

#### Korean Version of Mental Health Professionals Stress Scale

The MHPSS measures work stressors on seven subscales: “workload (WORKL),” “client-related difficulties (CRD),” “organizational structures and process (ORG),” “relationships and conflicts with other professionals (REC),” “lack of resources (RES),” “professional self-doubt (DOUBT),” and “home–work conflict (HWC).” Each subscale consists of six items, for a total of 42 items; the items are rated on a four-point Likert scale (0 = does not apply to me, 3 = does apply to me). The average total score and subscale scores were calculated, where higher scores indicated greater stress. The Cronbach's alpha of the total score in the original MHPSS was 0.87 for clinical psychologists and 0.94 for mental health nurses, showing good reliability (33).

#### Korean Version of Copenhagen Burnout Inventory

Concurrent validity of the K-MHPSS was tested against the CBI-K (39), which was developed to measure fatigue and burnout. The items are rated on a five-point Likert scale (0 = never/almost never or to a very low degree, 25 = seldom or to a low degree, 50 = sometimes or somewhat, 75 = often or to a high degree, 100 = always or to a very high degree), and subscale scores were used for analysis, where a higher score indicated a higher level of burnout. The scale consists of three subscales (personal burnout, work-related burnout, and client-related burnout), for a total of 19 items. The Korean version has been confirmed to have adequate reliability and validity (40). The Cronbach's alpha for the CBI-K subscales in this study were 0.91 for personal burnout, 0.82 for work-related burnout, and 0.90 for patient-related burnout.

#### Job Satisfaction Scale

Concurrent validity of the K-MHPSS was tested against the Job Satisfaction Scale developed by Park (41) based on the Job Satisfaction Scale items developed by Scarpello and Campbell (42). The scale consists of 25 items on satisfaction with the boss, work, compensation, colleagues, and work conditions, with each item rated on a seven-point Likert scale (1 = extremely dissatisfied, 7 = extremely satisfied) where higher scores indicate higher job satisfaction. The Cronbach's alpha in this study was 0.93, which was similar to that of the original scale (41).

#### Symptom Checklist-90-Revised

The SCL-90-R assesses overall psychological problems and psychopathological symptoms (43); the Korean version has been standardized (44). The items are rated on a five-point Likert scale (0 = not at all, 4 = extremely). A T score of 63 or higher for the global severity index (GSI) or 63 or higher for two or more major symptom dimensions is deemed clinically relevant (45). In this study, only the depression and anxiety scores were used to identify cut-off points for the K-MHPSS and examine relationships with stressors. The Cronbach's alpha in this study was 0.92 for depression and 0.91 for anxiety.

### Statistical Analysis

First, confirmatory factor analysis (CFA) was conducted to estimate the stability of the factor structure of the MHPSS in Korean mental health professionals. The original MHPSS had a seven-factor structure, but an Indian study of clinical psychologists (46) and a Korean study of counselors (38) identified a four-factor structure. Seven-factor and four-factor structures obtained from previous research (33, 38) were investigated *via* CFA. In addition, we conducted exploratory factor analysis (EFA) because this was the first attempt to use the MHPSS with clinical psychologists, nurses, and social workers in Korea, the potential for a cultural gap between Europe and Asia, and the inconsistency in the MHPSS factor structure in previous studies. Factors were extracted using the maximum likelihood method, and Geomin oblique rotation was used because of the possibility of correlations among the extracted factors. The number of factors was determined based on the Kaiser criterion (eigenvalues >1), RMSEA, and information criterion. Starting with a one-factor model, the number of factors was increased to select the model with a RMSEA of 0.05 or lower with the least number of factors (47) and lowest BIC (information criterion) (48). Only factors with at least three items and a factor loading >0.30 were retained (49, 50). Items loaded on two or more factors with a loading of 0.32 or higher (cross-loading) were deleted (51). Model fit indices where CFI and TLI were 0.90 or higher (52) and RMSEA and SRMR were 0.05 or lower (53) were considered to indicate a good model fit.

Concurrent validity was tested by analyzing the correlation between the K-MHPSS and the CBI-K and Job Satisfaction Scale. Cronbach's alpha for the entire scale and each subscale was calculated, and a value of 0.60 or higher was considered to indicate good reliability (54). Sensitivity and specificity were computed using receiver operating characteristics (ROC) curve analysis, and Youden's index was used to estimate the K-MHPSS cut-off points for depression and anxiety (55). Further, the area under the curve (AUC) was examined; AUC indicates the probability that the tool accurately classifies a randomly selected pair including a case with symptoms and a case without symptoms, that is, the tool's discriminatory ability. An AUC value of 0.70 or higher indicates fair discrimination (56, 57). Finally, between-group differences in the K-MHPSS total score and subscale scores were analyzed with one-way ANOVA, and the subfactor effects on depression and anxiety were analyzed using multiple regression. Considered the presence of gender differences in depression and anxiety (58, 59), gender was set as a control variable. Statistical analyses were performed using IBM SPSS version 22.0 and Mplus 8.5.

## Results

### Factor Structure

The result from the CFA models for each professional group could not demonstrate reasonable fit indices (see Table 2). It showed that current data could not replicate the factor structure of MHPSS. Therefore, we undertook an EFA by combining the three professional groups. Because it is recommended to conduct EFA with a large enough sample size (60), unlike CFA, which can perform with a relatively small sample size (61). To determine the number of factors, the fit indices were analyzed for a one-factor to a seven-factor model with an eigenvalue greater than one, and the seven-factor model (χ^2^ = 1,225.348, df = 588, *p* < 0.001, CFI = 0.935, TLI = 0.904, RMSEA = 0.044) was found to have the best fit for the data. However, there were items with loadings below 0.32 and cross-loaded items, so a second analysis was performed after deleting these eight items (1, 8, 14, 26, 33–35, 41). In the second analysis, both the six-factor model (χ^2^ = 766.338, df = 372, *p* < 0.001, CFI = 0.946, TLI = 0.918, RMSEA = 0.044, BIC = 46,803.148) and the seven-factor model (χ^2^ = 630.479, df = 344, *p* < 0.001, CFI = 0.961, TLI = 0.936, RMSEA = 0.039, BIC = 46,844.371) had good fit indices (Table 3); however, the seven-factor model had one factor with fewer than three items loaded, and considering the parsimony of the model, the model with fewer factors was deemed more appropriate. Hence, the six-factor model was selected.

**Table 2:** The CFA model fit indices for mental health professionals.

**Model**	****χ^2^****	**df**	**CFI**	**TLI**	**SRMR**	**RMSEA (90% C.I)**
**Clinical psychologist (** ***N*** **=** **200)**						
Seven-factor model	1,647.842^***^	798	0.761	0.742	0.093	0.073 (0.068–0.078)
Four-factor model	1,355.974^***^	588	0.711	0.691	0.099	0.081 (0.075–0.086)
**Nurse (** ***N*** **=** **157)**						
Seven-factor model	1,649.396^***^	798	0.747	0.727	0.092	0.082 (0.077–0.088)
Four-factor model	1,288.927^***^	588	0.735	0.716	0.087	0.087 (0.081–0.094)
**Social worker (** ***N*** **=** **201)**						
Seven-factor model	1,853.903^***^	798	0.699	0.675	0.091	0.081 (0.076–0.086)
Four-factor model	1,394.353^***^	588	0.705	0.684	0.090	0.083 (0.077–0.088)

*** *p < 0.001*.

**Table 3:** The EFA model fit indices for mental health professionals.

**Number of factors**	****χ^2^****	**df**	**CFI**	**TLI**	**SRMR**	**RMSEA (90% C.I)**	**BIC**
6	766.338^***^	372	0.946	0.918	0.028	0.044 (0.039–0.048)	46,803.148
7	630.479^***^	344	0.961	0.936	0.025	0.039 (0.034–0.043)	46,844.371

*** *p < 0.001*.

Factor 1 consisted of three items (4, 11, 25), with a loading range of 0.373–0.737, and was labeled “conflict with other professionals (COP).” Factor 2 consisted of ten items (3, 10, 17, 18, 24, 28, 31, 32, 38, 39), with a loading range of 0.307–0.788, and was labeled “organizational structure and processes (ORG).” Factor 3 consisted of four items (6, 13, 20, 27), with a loading range of 0.360–821, and was labeled “professional self-doubt (DOUBT).” Factor 4 consisted of seven items (7, 15, 21, 22, 29, 36, 42), with a loading range of 0.346–0.878, and was labeled “workload (WORKL).” Factor 5 consisted of six items (2, 9, 16, 23, 30, 37) with a loading range of 0.405–0.593 and was labeled “client-related difficulties (CRD).” Finally, Factor 6 consisted of four items (5, 12, 19, 40), with a loading range of 0.313–0.575, and was labeled “lack of resources (RES)” (Table 4). All items had factor loadings of 0.30 or higher for the six factors.

**Table 4:** Factor loading estimates after Geomin rotation and reliability.

	**Factor 1**	**Factor 2**	**Factor 3**	**Factor 4**	**Factor 5**	**Factor 6**
**Subscale I: conflict with other professionals (COP)**					
4. Conflict with other profession (e.g., doctor, nurse)	0.728					
11. Conflicting roles with other professional	0.737					
25. Criticism by other professional (e.g., doctor, nurse)	0.373					
**Subscale II: organizational structure and processes (ORG)**					
3. Lack of support from management		0.479				
10. Relationship with line manager		0.647				
17. Communications and flow of information at work		0.788				
18. Working in a multidisciplinary team		0.498				
24. Poor management and supervision		0.429				
28. Relationship with spouse/partner affects work		0.307				
31. The way conflicts are resolved in the organization		0.728				
32. Lack of emotional support from colleagues		0.572				
38. Organizational structure and policies		0.579				
39. Difficulty of working with certain colleagues		0.540				
**Subscale III: professional self-doubt (DOUBT)**					
6. Feeling inadequately skilled for dealing with emotional needs of clients/patients			0.774			
13. Uncertainty about own capabilities			0.821			
20. Feeling inadequately skilled for dealing with difficult clients/patients			0.798			
27. Doubt about the efficacy of therapeutic endeavors			0.360			
**Subscale IV: workload (WORKL)**					
7. Not enough time with family				0.681		
15. Not enough time to complete all tasks satisfactorily				0.584		
21. Taking work home				0.522		
22. Too many clients/patients				0.346		
29. Working too long hours				0.749		
36. Not enough time for recreation				0.878		
42. Inadequate time for friendships/social relationships				0.749		
**Subscale V: client-related difficulties (CRD)**					
2. Terminating with clients/patients					0.476	
9. Dealing with death or suffering					0.405	
16. No change or slowness of change in clients/patients					0.453	
23. Difficult and/or demanding clients/patients					0.539	
30. Physically threatening clients/patients					0.526	
37. Managing therapeutic relationships					0.593	
**Subscale VI: lack of resources (RES)**					
5. Lack of adequate staffing						0.313
12. Lack of financial resources for training courses/workshops						0.336
19. Shortage of adequate equipment/supplies						0.566
40. Poor physical working conditions						0.575
Cronbach's alpha	0.76	0.87	0.82	0.85	0.74	0.68
Eigenvalue	9.306	2.836	2.422	1.596	1.401	1.261

*The meaning of the gray values is factor loadings*.

### Descriptive Statistics

Table 5 shows the K-MHPSS scores and relevant evaluation scale scores for the entire sample. The mean K-MHPSS score was 1.25 (SD = 0.50), and the subscale scores ranged from 0.94 to 1.46, with the lowest mean score for COP and highest mean score for DOUBT. The absolute values of skewness and kurtosis did not exceed one, and thus the assumption of normal distribution does not seem to be violated (62).

**Table 5:** Descriptive statistics for the K-MHPSS and other related measurements.

	**Mean**	**SD**	**Skewness**	**Kurtosis**
MHPSS Total	1.25	0.50	0.165	0.151
MHPSS- COP	0.94	0.76	0.651	−0.236
MHPSS-ORG	1.25	0.64	0.134	−0.374
MHPSS-DOUBT	1.46	0.72	0.210	−0.478
MHPSS-WORKL	1.20	0.74	0.324	−0.682
MHPSS-CRD	1.25	0.62	0.166	−0.403
MHPSS-RES	1.34	0.72	0.172	−0.627
CBI-K personal	48.14	22.40	0.013	−0.752
CBI-K work-related	43.66	21.26	0.231	−0.671
CBI-K Client-related	32.63	22.32	0.623	−0.184
Job satisfaction	3.97	0.94	0.092	0.135
SCL-90-R Depression	44.97	10.79	0.342	−0.326
SCL-90-R Anxiety	44.46	9.33	0.778	0.422

### Concurrent Validity

The correlation between the K-MHPSS and other scales was analyzed using Pearson product-moment correlation to determine the K-MHPSS's concurrent validity. The K-MHPSS score was significantly positively correlated with the personal (*r* = 0.62, *p* < 0.01), work-related (*r* = 0.62, *p* < 0.01), and client-related (*r* = 56, *p* < 0.01) CBI-K factors, and significantly negatively correlated with the total job satisfaction score (*r* = −0.46, *p* < 0.01). Table 6 shows the correlations between the K-MHPSS total score and subscale scores and other relevant scales.

**Table 6:** Correlation between the K-MHPSS and other related measurements.

		**1**	**2**	**3**	**4**	**5**	**6**	**7**	**8**	**9**	**10**	**11**	**12**	**13**
1	MHPSS Total	1												
2	MHPSS- COP	0.64^**^	1											
3	MHPSS-ORG	0.84^**^	0.56^**^	1										
4	MHPSS-DOUBT	0.56^**^	0.27^**^	0.34^**^	1									
5	MHPSS-WORKL	0.69^**^	0.24^**^	0.39^**^	0.24^**^	1								
6	MHPSS-CRD	0.74^**^	0.45^**^	0.49^**^	0.46^**^	0.36^**^	1							
7	MHPSS-RES	0.73^**^	0.44^**^	0.56^**^	0.29^**^	0.45^**^	0.48^**^	1						
8	CBI-K personal	0.62^**^	0.29^**^	0.48^**^	0.29^**^	0.62^**^	0.41^**^	0.40^**^	1					
9	CBI-K work-related	0.62^**^	0.33^**^	0.48^**^	0.34^**^	0.59^**^	0.40^**^	0.40^**^	0.81^**^	1				
10	CBI-K Client-related	0.56^**^	0.32^**^	0.38^**^	0.42^**^	0.40^**^	0.53^**^	0.37^**^	0.54^**^	0.65^**^	1			
11	Job satisfaction	−0.46^**^	−0.24^**^	−0.53^**^	−0.21^**^	−0.28^**^	−0.17^**^	−0.40^**^	−0.37^**^	−0.46^**^	−0.28^**^	1		
12	SCL-90-R Depression	0.42^**^	0.23^**^	0.36^**^	0.30^**^	0.33^**^	0.24^**^	0.26^**^	0.51^**^	0.51^**^	0.38^**^	−0.28^**^	1	
13	SCL-90-R Anxiety	0.41^**^	0.28^**^	0.35^**^	0.29^**^	0.31^**^	0.25^**^	0.23^**^	0.46^**^	0.45^**^	0.37^**^	−0.22^**^	0.82^**^	1

** *p < 0.01*.

### Reliability

The internal consistency of the K-MHPSS was examined based on Cronbach's alpha for the total score and subscale scores for the entire sample (Table 4). Cronbach's alpha was 0.92 for the total score (34 items), 0.76 for COP, 0.87 for ORG, 0.82 for DOUBT, 0.85 for WORKL, 0.74 for CRD, and 0.68 for RES, showing acceptable consistency for RES and good consistency for the remaining subscales.

### Cut-Off Point

**Figure 1:**
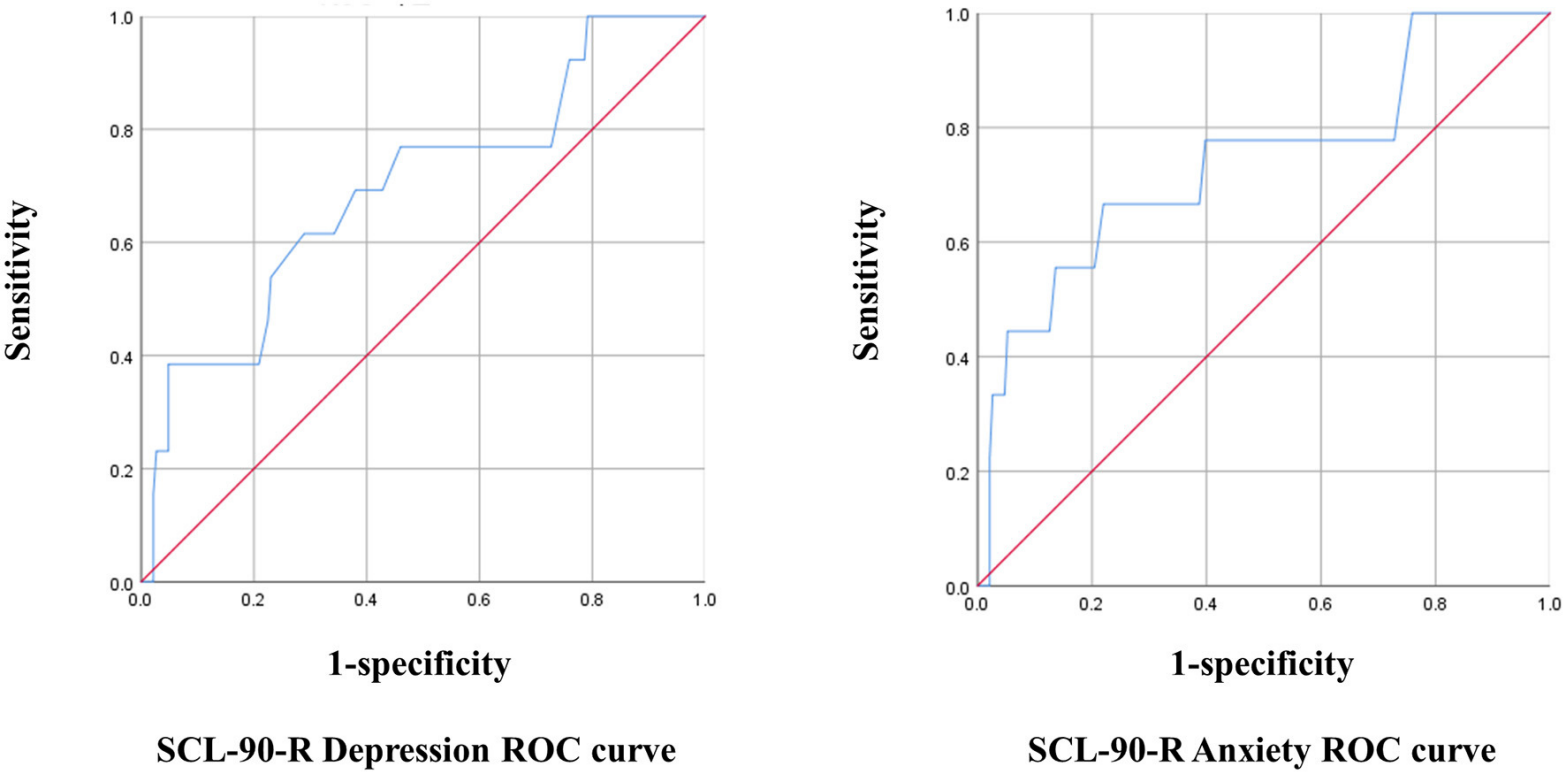
ROC curve for clinical psychologist.

**Figure 2:**
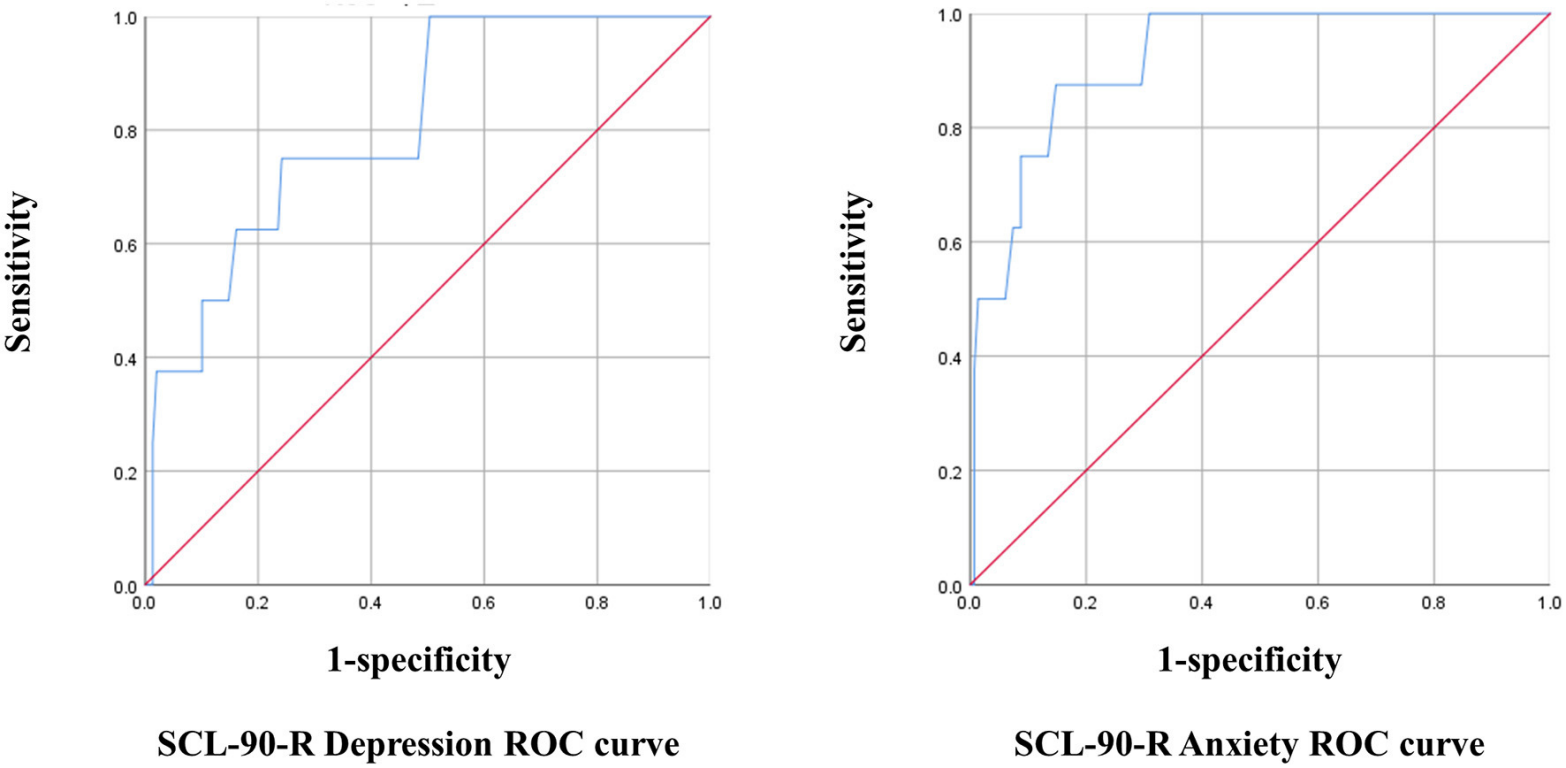
ROC curve for nurse.

**Figure 3:**
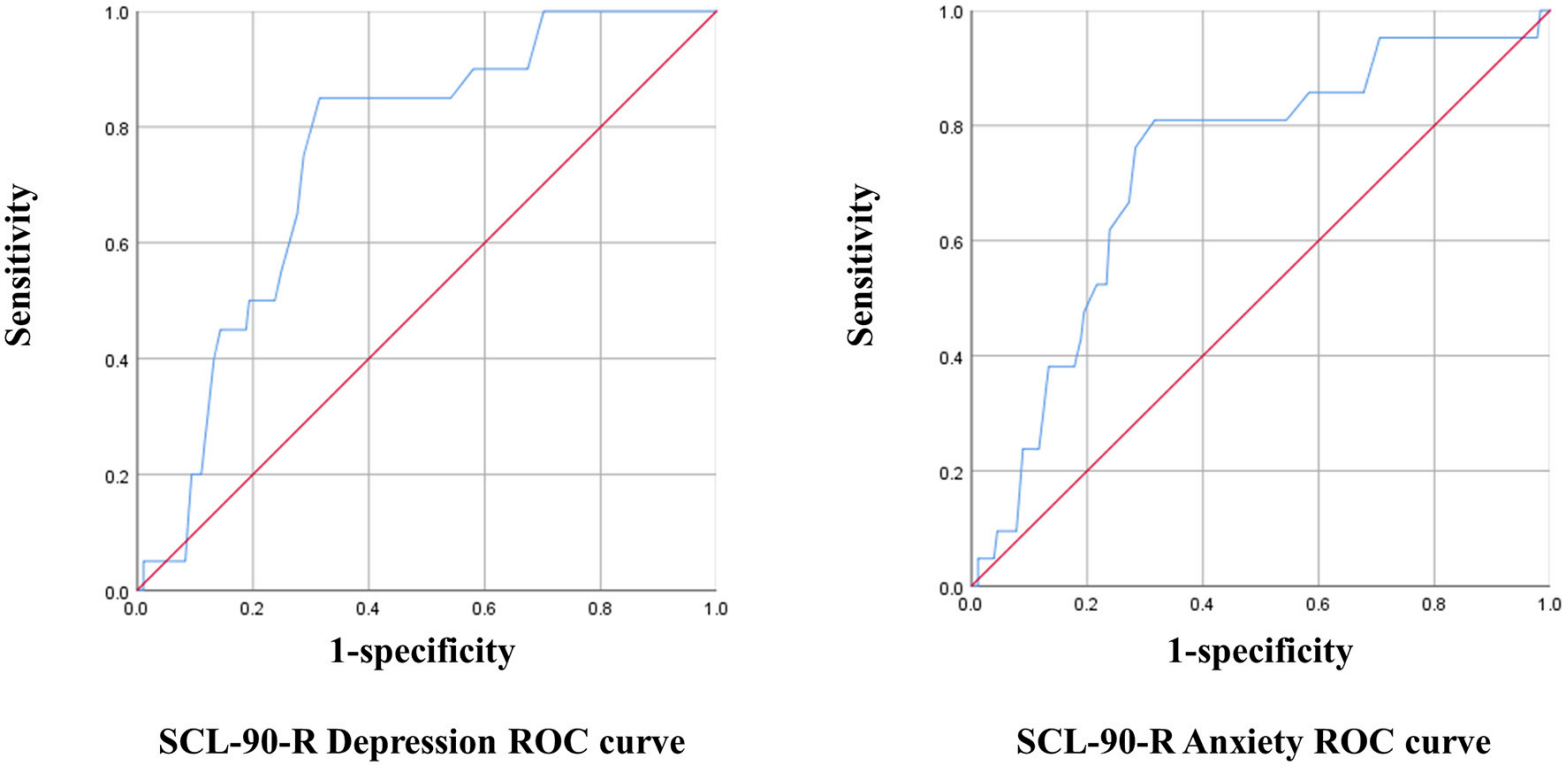
ROC curve for social worker.

The optimal cut-off point for the K-MHPSS was estimated in each mental health professional group using the depression and anxiety subscales of the SCL-90-R. The cut-off point for diagnosing clinical depression and anxiety in Koreans is 63T, which is equivalent to that used in the United States. With 63T as the reference, cases with a score of 63 or higher were considered to have symptoms, and cases with a score below 63 were considered to have no symptoms for the ROC analysis. In clinical psychologist, the K-MHPSS cut-off point for depression was 1.78, with a sensitivity of 38% and specificity of 95% (Youden's Index = 0.34); the cut-off point for anxiety was 1.45, with a sensitivity of 67% and specificity of 78% (Youden's Index = 0.45). In nurses, the K-MHPSS cut-off point for depression was 1.66, with a sensitivity of 75% and specificity of 76% (Youden's index = 0.51); the cut-off point for anxiety was 1.78, with a sensitivity of 88% and specificity of 85% (Youden's index = 0.73). In social workers, the K-MHPSS cut-off point for depression was 1.46, with a sensitivity of 85% and specificity of 69% (Youden's index = 0.54); the cut-off point for anxiety was 1.46, with a sensitivity of 81% and specificity if 68% (Youden's index = 0.49). Figures 1–3 and Table 7 show the ROC curve, AUC, and other applicable data for the MHPSS. The values of AUC were ranged from 0.696 to 0.810 for depression and ranged from 0.725 to 0.922 for anxiety in each profession, showing that the K-MHPSS had good discrimination for depression and anxiety (57).

**Table 7:** AUC, standard error, 95% confidence interval, and *p*-value.

	**AUC**	**SE**	**95% Confidence interval**		**Significance**
			**Lower**	**Upper**	
**Clinical psychologist**					
Depression	0.696	0.08	0.539	0.854	<0.05
Anxiety	0.740	0.10	0.553	0.928	<0.05
**Nurse**					
Depression	0.810	0.07	0.672	0.947	<0.01
Anxiety	0.922	0.04	0.849	0.994	<0.001
**Social worker**					
Depression	0.752	0.05	0.658	0.845	<0.001
Anxiety	0.725	0.06	0.613	0.838	<0.01

### Comparison

As shown in Table 8, there were between-group differences for the K-MHPSS total score and subscale scores. The data for WORKL did not satisfy the homogeneity of variance assumption, so Welch's F test and Dunnett' T3 *post hoc* comparison were performed. The remaining factors all satisfied the homogeneity of variance assumption, so Scheffe's *post hoc* comparison was performed. There was a significant between-group difference in the K-MHPSS total score [*F*_(2, 555)_ = 11.27, *p* < 0.001], and *post hoc* tests revealed that nurses and social workers had significantly higher scores than clinical psychologists. For the subscale scores, there were between-group differences for all subscale scores except the WORKL score. *Post hoc* tests showed that nurses and social workers had significantly higher COP [*F*_(2, 555)_ = 16.18, *p* < 0.001] and ORG [*F*_(2, 555)_ = 17.05, *p* < 0.001] scores than clinical psychologists, and social workers had significantly higher DOUBT scores [*F*_(2, 555)_ = 4.36, *p* < 0.05] than nurses. Nurses and social workers had significantly higher CRD [*F*_(2, 555)_ = 14.73, *p* < 0.001] scores than clinical psychologists, and nurses had significantly higher RES [*F*_(2, 555)_ = 7.81, *p* < 0.001] scores than clinical psychologists.

**Table 8:** Analysis of variance for mental health professionals.

	**Clinical psychologist^**a**^**	**Nurse^**b**^**	**Social worker^**c**^**	***F*_**(2, 555)**_**	***Post hoc***
**MHPSS**	***M* (SD)**	***M* (SD)**	***M* (SD)**		
Total	1.12 (0.46)	1.35 (0.54)	1.29 (0.48)	11.27^***^	a < b, c
COP	0.71 (0.68)	1.14 (0.73)	1.02 (0.80)	16.18^***^	
ORG	1.05 (0.61)	1.42 (0.64)	1.31 (0.62)	17.05^***^	
DOUBT	1.44 (0.72)	1.34 (0.68)	1.56 (0.75)	4.36^*^	b < c
WORKL	1.22 (0.81)	1.25 (0.75)	1.24 (0.66)	0.94	–
CRD	1.07 (0.57)	1.38 (0.65)	1.34 (0.61)	14.73^***^	a < b, c
RES	1.20 (0.74)	1.49 (0.72)	1.37 (0.67)	7.81^***^	a < b

* *p < 0.05*,

*** *p < 0.001*.

*COP, conflicts with other professionals; ORG, organizational structure and processes; DOUBT, professional self-doubt; WORKL, workload; CRD, client-related difficulties; RES, lack of resources*.

*Given that the homogeneity of variance assumption was violated for the WORKL, we also reported the result of Welch's F test followed by Dunnett's T3 post hoc comparisons. For WORKL Welch's F = 1.044, df = 2, 355.1, p = 0.353*.

### Regression Analysis

The effects of the K-MHPSS sub-factors of depression and anxiety in clinical psychologists, nurses, and social workers were analyzed with multiple regression (Tables 9, 10). Model 1 included gender as control variable, and Model 2 added subscale scores as independent variables. The results showed that after controlling gender, depression predictors included ORG (β = 0.267, *p* < 0.01) and WORKL (β = 0.181, *p* < 0.05) in clinical psychologists, DOUBT (β = 0.238, *p* < 0.01) and WORKL (β = 0.207, *p* < 0.05) in nurses, and ORG (β = 0.389, *p* < 0.001), DOUBT (β = 0.168, *p* < 0.05), and WORKL (β = 0.214, *p* < 0.01) in social workers. Anxiety predictors were DOUBT (β = 0.283, *p* < 0.01) and WORKL (β = 0.315, *p* < 0.01) in nurses and ORG (β = 0.379, *p* < 0.001) and WORKL (β = 0.219, *p* < 0.01) in social workers after controlling gender. There were no significant anxiety predictors in clinical psychologists.

**Table 9:** Multiple regression analysis with depression as the dependent variable.

		**Clinical psychologist**		**Nurse**		**Social worker**	
		**β**	***t* (*p*)**	**β**	***t* (*p*)**	**β**	***t* (*p*)**
Model 1	Gender	−0.092	−1.305 (0.194)	−0.129	−1.623 (0.107)	0.018	0.253 (0.800)
	*R* ^2^	0.009		0.017		0.000	
	*F*	1.702		2.635		0.064	
Model 2	Gender	−0.126	−1.896 (0.059)	−0.112	−1.546 (0.124)	0.009	0.145 (0.855)
	COP	0.015	0.178 (0.859)	0.122	1.323 (0.188)	−0.022	−0.297 (0.767)
	ORG	0.267	2.939 (0.004)	−0.147	−1.353 (0.178)	0.389	4.988 (0.000)
	DOUBT	0.126	1.677 (0.095)	0.238	2.776 (0.006)	0.168	2.377 (0.018)
	WORKL	0.181	2.498 (0.013)	0.207	2.063 (0.041)	0.214	3.157 (0.002)
	CRD	0.005	0.059 (0.953)	0.088	0.929 (0.354)	−0.116	−1.517 (0.131)
	RES	−0.031	−0.376 (0.707)	0.087	0.851 (0.396)	0.045	0.594 (0.553)
	*R* ^2^	0.169		0.237		0.324	
	*F*	5.570^***^		6.599^***^		13.202^***^	

*Dependent variable: SCL-90-R depression*

*** *p < 0.001*.

*COP, conflicts with other professionals; ORG, organizational structure and processes; DOUBT, professional self-doubt; WORKL, workload; CRD, client-related difficulties; RES, lack of resources*.

**Table 10:** Multiple regression analysis with anxiety as the dependent variable.

		**Clinical psychologist**		**Nurse**		**Social worker**	
		**β**	***t* (*p*)**	**β**	***t* (*p*)**	**β**	***t* (*p*)**
Model 1	Gender	−0.078	−1.107 (0.270)	−0.069	−0.865 (0.388)	0.04	0.567 (0.571)
	*R* ^2^	0.006		0.005		0.005	
	*F*	1.225		0.748		0.321	
Model 2	Gender	−0.101	−1.483 (0.140)	−0.051	−0.729 (0.467)	0.026	0.418 (0.676)
	COP	0.16	1.903 (0.058)	0.131	1.465 (0.145)	0.071	0.939 (0.349)
	ORG	0.119	1.287 (0.200)	−0.125	−1.195 (0.234)	0.379	4.734 (0.000)
	DOUBT	0.119	1.547 (0.123)	0.283	3.417 (0.001)	0.078	1.07 (0.286)
	WORKL	0.135	1.827 (0.069)	0.315	3.253 (0.001)	0.219	3.145 (0.002)
	CRD	0.08	0.931 (0.353)	0.076	0.835 (0.405)	−0.151	−1.918 (0.057)
	RES	−0.103	−1.213 (0.227)	−0.028	−0.283 (0.778)	0.026	0.335 (0.738)
	*R* ^2^	0.134		0.288		0.285	
	*F*	4.235^***^		8.627^***^		11.009^***^	

*Dependent variable: SCL-90-R anxiety*

*** *p < 0.001*.

*COP, conflicts with other professionals; ORG, organizational structure and processes; DOUBT, professional self-doubt; WORKL, workload; CRD, client-related difficulties; RES, lack of resources*.

## Discussion

This study standardized the K-MHPSS for use with mental health professionals in Korea, including clinical psychologists, nurses, and social workers. The study's first objective was to test the validity and reliability of the K-MHPSS using factor analysis, correlation analysis with relevant scales, and internal consistency testing for the K-MHPSS total score and subscale scores. We tested the factor structure obtained from prior research *via* CFA to Korean mental health professionals but did not show an acceptable level of fit indices. A subsequent EFA revealed that a six-factor structure could be appropriate in Korean mental health professionals. However, construct validity could not be confirmed because CFA for the six-factor structure did not conduct. The K-MHPSS's total score and subscale scores were significantly positively correlated with the personal, work-related, and client-related burnout scales in the CBI-K and significantly negatively correlated with the Job Satisfaction Scale, thereby confirming good concurrent validity. Moreover, the Cronbach's alpha for the K-MHPSS total score was 0.92, and that for the subscale scores ranged from 0.68 to 0.87, showing good reliability overall.

The K-MHPSS six-factor structure was found to have the best fit, which contrasts with the original scale's seven-factor structure and the four-factor structure found to be optimal in a study that validated the MHPSS with Korean counselors (38), and a study that examined the factor structure for MHPSS use with Indian clinical psychologists (46). The difference in the factor structures may be attributable to the sample size and characteristics. Costello and Osborne (49) suggested that the likelihood of misclassifying an item with the wrong factor increases with small samples, which then undermines generalizability and replicability, and that the sample size should be close to 20 times the number of items for highest accuracy. In Cushway et al.'s (33) study of 154 clinical psychologists and 111 nurses, the original seven-factor structure was found to be the optimal structure for clinical psychologists, but only six of the seven factors were effective, with the remaining items included in a new factor (“shortage of time”) for nurses. An MHPSS study of clinical psychologists in India had only 116 participants, which may explain differences in our study results, where factor analysis was conducted on 558 mental health professionals. A study of counselors in Korea had a larger sample size compared to previous studies, but the organization characteristics and type of work performed by counselors differed from that of the mental health professionals who participated in this study, which might have led to the difference in the factor structure.

In this study, the six factors obtained through EFA were labeled similar to the original scale: conflict with other professionals, organizational structure and process, professional self-doubt, workload, client-related difficulties, and lack of resources. The first factor, COP, was equivalent to REC in the original scale, but items 18, 32, and 39 from the six items included in the original study's REC loaded on ORG in this study. In a study of Korean counselors, the ORG and REC items were merged to form new factors “organization-related difficulties” and “relationship conflicts” (38), and similarly, the items related to conflict with other professionals were classified as COP, while items related to relationships with colleagues were classified as ORG in this study.

Among the six HWC items in the original scale, items 7, 21, and 42 loaded on WORKL, while item 28 loaded on ORG. Items 14 and 35 were not loaded on a single factor and thus were removed. Choi and Lee (38) also had items 7, 21, and 42 loaded on WORKL, presumably because of cultural differences. Kim and Kim (63) investigated factors affecting work-family conflict experienced by married hospital workers, including health care professionals, and found that factors related to workload and work burden, such as overtime and unpredictability of work, contributed to work-family conflict. While all participants were married, only those with a child age six or younger had adverse impacts on work-family conflict. Other factors related to family roles were not associated with work-family conflict. Based on these results, we can predict that work-family conflict and workload are similar and closely linked concepts, which may have led to items 7 (“not enough time with family”), 21 (“taking work home”), and 42 (“inadequate time for friendships/social relationships”) loading on workload, while items 14 (“inability to separate personal from professional role”) and 35 (“work emphasizes feelings of emptiness and/or isolation”) were not classified into a specific factor because they are not largely relevant to work-family conflict as perceived by Koreans.

The second objective of this study was to present the optimal K-MHPSS cut-off points for initial clinical depression and anxiety screening. The K-MHPSS cut-off points for depression and anxiety were 1.78 and 1.45 in clinical psychologists, 1.66 and 1.78 in nurses, and 1.46 and 1.46 in social workers. However, they could not be compared with that of the original scale and other studies, because cut-off points were not reported. The cut-off points were higher than the mean scores for each group, with 7.0 and 22.0% of clinical psychologists, 26.2 and 17.9% of nurses, and 36.8% of social workers showing scores above the cut-off points for depression and anxiety, respectively. The K-MHPSS had good discriminatory power for depression and anxiety; however, since this study was conducted on general mental health professionals and not a clinical patient group diagnosed with depression and anxiety-related disorders, using the K-MHPSS as the primary screening tool for clinical patients may have limitations. Although stress is not a disease in itself, it can lead to psychological problems, including depression and anxiety. Therefore, the K-MHPSS may be used as the initial screening tool for mental health professionals, and prophylactic interventions could be provided to professionals exhibiting stress that exceeds the cut-off points

The study's third objective was to examine differences in the stressors experienced by the type of profession. Nurses and social workers showed significantly higher total, COP, ORG, and CRD scores as compared to clinical psychologists, and nurses showed significantly higher RES scores than clinical psychologists. Cushway et al. (33) also reported that nurses showed significantly higher scores on the same subscales compared to clinical psychologists. Moreover, one study that administered the MHPSS to 108 social workers and compared the results with Cushway et al.'s clinical psychologist and nurse scores, reported higher total, REC, ORG, and CRD scores among social workers as compared to clinical psychologists, which was consistent with our results, although it is not known whether the differences were significant (30).

The lower COP and ORG scores in clinical psychologists may result from clinical psychologists working alone because of the nature of their work. Clinical psychologists who work in a medical institution, practice or work as a freelancer in a private treatment facility, rarely engage in activities within the organization or interact with other professionals because their major work is providing therapy, which may have resulted in lower scores for the corresponding stressor as compared to other professions. Further, nurses and social workers had significantly higher CRD scores than clinical psychologists. Jobs involving patients/clients may pose greater stressors for nurses and social workers despite that all three professions deal directly with patients, which could be because nurses spend most of their time with patients and social workers manage numerous cases and provide wide-ranging services. Among the three professions, nurses reported the highest RES score. The number of mental health nurses per 100,000 population in Korea is 14.7, which is more than twice as few as in high-income countries (64), and such short staffing may have contributed to the high RES score among mental health nurses.

Regression analysis was performed for each group to examine the effects of stressors on depression and anxiety with controlling gender. In all three groups, WORKL was a significant predictor of depression or anxiety. Past studies have also reported that excessive workload predicts depression and anxiety in healthcare providers, including nurses, as well as in general office workers (65–67), showing consistent findings among mental health professionals as well. Another factor affecting depression and anxiety in mental health professionals was DOUBT. A previous study argued that professional self-doubt is the greatest stressor for clinical psychologists because they sense limitations in therapy effectiveness and their competence as therapists (23). However, although clinical psychologists in our study had DOUBT scores that were higher than their other MHPSS subscale scores, DOUBT did not predict their depression or anxiety. In contrast, DOUBT was a significant predictor of depression or anxiety among nurses and social workers. Professional self-doubt specifically addresses characteristic mental health-related job descriptions, so studies examining its effect on psychological problems in mental health professionals are rare. However, given the finding that excessive workload increases emotional distress and reduces professional efficacy in nurses (65), incompetency in professional tasks may lead to professional self-doubt, which in turn is predicted to adversely impact professionals' mental health. Further studies are needed to examine the relationship between stressors and psychological problems in mental health professionals.

This study has a few limitations. First, although the K-MHPSS with a six-factor structure was appropriate to Korean mental health professionals, the construct validity could not be confirmed since the CFA for this structure has not been performed. Several previous research randomly divided samples into two equal parts and conducted EFA and CFA, respectively, but this method requires a large enough sample size (68–70). The sample size in this study was not suitable for the cross-validation method. Therefore, subsequent studies should verify the six-factor structure derived from this study with mental health professionals, including clinical psychologists, nurses, and social workers. Second, self-report questionnaires were used, so there is a potential for underreporting or biased responses relating to social desirability. In addition, the study parameters were measured cross-sectionally, so causality between stressors and psychological symptoms could not be examined. Thus, subsequent studies should address the limitations of the questionnaire and study design. Third, study participants were mental health professionals as recognized by the Korea Ministry of Health and Welfare, including clinical psychologists, nurses, and social workers. However, various other professionals also provide mental health services to patients and clients, including school psychologists, and occupational therapists. Subsequent studies should examine the reliability and validity of the K-MHPSS in other types of mental health professionals that were not included in this study.

Despite these limitations, this study provides evidence for the reliability and validity of the K-MHPSS for use with clinical psychologists, nurses, and social workers. Moreover, the results which identify the difference in stressors experienced among the professionals and the relationships between the specific stressors and depression and anxiety give knowledge for establishing an effective intervention approach. As there are few or no studies to verify the effectiveness of stress management intervention for mental health professionals (71), exploring interventions primarily based on the stressors specific to each mental health profession with reference to our findings would contribute to promoting mental health professionals' health and thereby facilitating quality mental health service to patients.

## Data Availability Statement

The datasets presented in this article are not readily available because the participants did not consent to share their raw data. Requests to access the datasets should be directed to Subin Park, subin21@korea.kr.

## Ethics Statement

The studies involving human participants were reviewed and approved by National Center for Mental Health. The patients/participants provided their written informed consent to participate in this study.

## Author Contributions

EL, VR, and JL conceived and designed the study. EL, JL, and HHo collected data. EL, HHo, and HHa contributed to performing statistical analysis and interpreting the data. EL and HHo wrote the manuscript. VR and SP reviewed critically and revised the manuscript. All authors contributed to the article and agreed to be accountable for the content of the final version of the manuscript.

## Conflict of Interest

The authors declare that the research was conducted in the absence of any commercial or financial relationships that could be construed as a potential conflict of interest.

## Publisher's Note

All claims expressed in this article are solely those of the authors and do not necessarily represent those of their affiliated organizations, or those of the publisher, the editors and the reviewers. Any product that may be evaluated in this article, or claim that may be made by its manufacturer, is not guaranteed or endorsed by the publisher.
